# Nutritional knowledge as a determinant of vitamin and mineral supplementation during pregnancy

**DOI:** 10.1186/1471-2458-13-1105

**Published:** 2013-12-01

**Authors:** Alina D Popa, Otilia Niţă, Lidia I Graur (Arhire), Raluca M Popescu, Gina E Botnariu, Laura Mihalache, Mariana Graur

**Affiliations:** 1 Nursing Department, University of Medicine and Pharmacy “Gr. T. Popa”, Iaşi, România; 2 Diabetes, Nutrition and Metabolic Diseases Department, University of Medicine and Pharmacy “Gr. T. Popa”, Iaşi, România

**Keywords:** Pregnancy, Mineral supplement, Vitamin supplements, Folic acid, Iron

## Abstract

**Background:**

Pregnancy is a critical period for both woman and baby from a nutritional perspective. Nutritional education is considered an important tool for promoting a healthy lifestyle, but has not been studied as a determinant for maternal use of supplements during pregnancy, especially in Romania, where evidence about pregnancy and nutrition is scarce. This study aimed to evaluate the relationship between nutritional knowledge and the use of folic acid, iron and multivitamin supplements during pregnancy and to assess the influence of socio-demographic factors and prenatal care.

**Methods:**

We conducted a cross-sectional study on a sample of 400 pregnant women admitted to the Cuza-Vodă Obstetrics and Gynaecology Clinical Hospital in Iaşi, Romania, during August-September 2010. We collected self-reported data regarding socio-demographic characteristics, number of prenatal check-ups and the use of folic acid, iron and multivitamin supplements during pregnancy. We assessed nutritional knowledge using a standardized questionnaire divided into three sections: general nutritional recommendations for pregnant women; the roles of nutrients; and sources of nutrients. We used logistic regression to analyse the associations between these factors.

**Results:**

The prevalence of the use of supplements during pregnancy was 48% for folic acid, 45.3% for iron and 68% for multivitamins. Above-average nutritional knowledge was independently associated with the use of folic acid (aOR, 4.7; 95% CI, 1.6-13.8), iron (aOR, 2.6; 95% CI, 1.2-5.7) and multivitamins (aOR, 2.8; 95% CI, 1.2-6.8). The use of folic acid was independently associated with a higher level of formal education (aOR, 5.2; 95% CI, 2.1-12.8) and an early start in prenatal care (aOR, 3.4; 95% CI, 1.0-11.1). Women with a higher education (aOR, 2.3; 95% CI, 1.1-4.9), more than 10 prenatal visits (aOR, 7.2; 95% CI, 3.4-15.0) and those who received advice on breastfeeding (aOR, 2.0; 95% CI, 1.1-3.5) were more likely to use iron during pregnancy. Similar results were found when analysing the contributing factors for the use of multivitamins: more than 12 years of schooling (aOR, 3.4; 95% CI, 1.4-7.9) and appropriate prenatal care (aOR, 9.4; 95% CI, 4.5-19.5).

**Conclusions:**

Level of nutritional knowledge has a strong independent association with the use of supplements during pregnancy.

## Background

Adequate nutrition during pregnancy is associated with optimal foetal growth [1,2], normal pregnancy duration [3] and a decrease in the risk of congenital abnormalities [4]. A lack of vitamins and minerals can adversely influence the newborn’s weight, even if the dietary intake of proteins and energy is adequate [5,6]. The results of several meta-analyses reinforce the beneficial role of iron and folic acid supplements during pregnancy in reducing the prevalence of neural tube defects, low-birth weight babies, small for gestational age newborns and maternal anaemia [7-9]. Some cross-sectional studies show the same positive results for the use of multivitamins [10], but there is still debate as to whether these are superior to iron and folic acid [11].

Anaemia during pregnancy is a public health problem that affects both developed and developing countries and has an impact not only on health providers, but also on the socio-economic environment [12]. Prevention strategies for iron deficiency and maternal anaemia include nutritional education, promoting the intake of iron and folic acid supplements, and food fortification [13]. Daily use of iron supplements is effective when administered under supervision in clinical interventional trials, but is less so in public health programmes because of difficulty engaging with the target population. Lack of engagement may be a consequence of insufficient involvement of policy makers, issues regarding distribution and costs and difficulties in training the educators, or the beliefs and cultural practices of the target population and the characteristics and side effects of supplements [11,14]. In Romania, a study conducted by the Alfred Rusescu Institute of Mother’s and Child’s Care (IOMC), found that the use of iron supplements during pregnancy in Romania only reduced the incidence of anaemia at birth by 5% even when iron supplements were provided free of charge under reimbursement arrangements from 16 weeks of gestation, and treatment was supervised by primary care physicians [14].

Several studies emphasize the importance of the relationship between iron, folic acid or multivitamin intake and socio-demographic factors such as age, level of education, area of residence, marital status, parity, economical status and characteristics of prenatal care (including gestational age at the beginning of prenatal care and the number of prenatal consultations) [15-19]. More frequent use of supplements during pregnancy was found to be associated with a higher socio-economic status, higher level of education and use of prenatal medical services in studies conducted in Finland [15], the United Kingdom [16], the United States of America [17], Denmark [18] and Tanzania [19]. One study conducted in Poland concluded that the level of nutritional knowledge of pregnant women regarding mineral and vitamins was insufficient, without focusing on the relationship with their intake [20]. Another study showed that a higher level of nutritional knowledge was associated with vitamin/mineral supplementation use [21]. In Romania it is not clear whether there is a relationship between socio-demographic factors or the level of nutritional knowledge and the use of iron, folic acid and multivitamins in pregnancy.

The aim of this study was to assess the determinants of the use of folic acid, iron and multivitamins supplements by expectant mothers in Romania, in terms of nutritional knowledge evaluated by a structured questionnaire, socio-demographic factors and engagement with prenatal care.

## Methods

### Design and study population

We conducted a cross-sectional study during August and September 2010 on a sample of 400 consecutive women who had given birth in a regional specialist obstetric centre, the Cuza-Vodă Clinical Hospital in Iaşi, Romania.

We invited women aged between 15 and 49 years, resident in Iaşi county, with singleton pregnancies to participate in the study within 48–72 hours of giving birth. We excluded stillbirths, those who declined to participate, those with obstetric pathology, or psychiatric, cognitive or other disease that might adversely affect understanding of the study objectives or the ability to provide accurate information. Those who did not complete the interview were recorded as having refused to participate and were taken into account when calculating the refusal rate, but excluded from the final analysis.

Sample size calculation was undertaken using the single proportion formula [22]. We selected a 95% confidence level and an assumed prevalence of supplement use of 50%, informed by other studies [15-19,22], due to the lack of prior data in our region. It is the single proportion formula (n = [Z α / 2] 2 P (1-p] / d2) at 95% confidence interval, where Z α / 2 = 1.96, P = prevalence of 50%, and d = 5% of marginal error [22]. We also defined a reference group to estimate the sample size. Owing to the lack of other available data, we used the annual number of live births in Iaşi county reported on 01 July 2009 (n = 9,499) to define the size of the reference group [23]. Based on these calculations, the minimum sample size required was 370. A number of 410 women was invited to participate at the study. From the final analysis ten questionnaires were excluded representing drop-outs (refusals after the initial moment of the study or incomplete questionnaires). Thus, the rate of refusal was relatively low, of only 2.5% of all the women invited to participate in the study.

### Data collection

We created a *pro forma* for data collection that was completed during an interview conducted 48 to 72 hours after delivery. In Romania, almost all women are hospitalised for at least 3 days post-partum during which time the newborn is immunised against tuberculosis and hepatitis B; it is very uncommon to leave the hospital earlier. We collected:

•Demographic data: age, area of residence (urban or rural), family status (married or single, wanted or unwanted pregnancy, parity), and duration of formal education;

•Engagement with prenatal care: gestational age at the beginning of care, provider of care (general practitioner or obstetrician), and total number of visits. The latter were subdivided into three categories according to current recommendations: ≤4 visits (inadequate), 5–9 visits (intermediate), ≥10 visits (adequate) [14];

•Components of prenatal care: advice given on breastfeeding and diet (yes/no answers);

•Use of oral supplements: including folic acid, iron and multivitamins (yes/no answers to each), and the trade names of supplements used.

### Development and validation of the nutritional knowledge questionnaire

To evaluate nutritional knowledge we developed a questionnaire based on the recommendations of the “Pregnancy Notebook” [24] and “The Principles of the Child’s and Mother’s Diet: Guidelines for Health Providers at Community Level” [25], educational tools developed by the Ministry of Health in Romania. A draft list of 40 questions was revised by a group of experts. These were divided into four categories and concerned knowledge of general dietary recommendations during pregnancy, the sources of nutrients, the roles of nutrients during pregnancy, and the importance of breastfeeding. Participants could respond “yes”/“no”/“don’t know” to each question. After expert review, the final questionnaire used had 29 items (see Additional file 1).

To validate the nutritional knowledge questionnaire, item analysis was performed to determine item difficulty and item discrimination indices to increase reliability. The items that were too difficult, too easy, or that were found to have near-zero or negative discrimination were eliminated. As a result, 26 items were included in the final analysis. The Cronbach’s α coefficient was acceptable for all sections (general recommendations, 0.66; sources of nutrients, 0.77; the role of nutrients, 0.77), except for breastfeeding (0.60), which was therefore eliminated. For the remaining 19 questions, the internal fidelity was satisfactory (the correlation coefficient between the two halves - 0.83; the-split half reliability - 0.91; Guttman split-half reliability coefficient- 0.91 and Cronbach’s α coefficient - 0.88) [26]. Construction validity was also found to be satisfactory (ANOVA F statistic = 39.9, p < .001), and *post hoc* testing revealed statistically significant differences between the mean score of the questionnaire according to each category of formal education (4.9 for 1–4 years of formal education; 8.35 for 5–8 years of education; 10.8 for 9–12 years of education; and 13.7 for more than 12 years; p < .001).

Based on these scores, we created the following categories of nutritional knowledge: low level (<mean – standard deviation), medium level (mean ± standard deviation) and high level (>mean + standard deviation). The mean score was 11.0 (95% CI, 10.5-11.5) and standard deviation was 4.8 (Table 1).

**Table 1:** Background characteristics of the participants

**Background characteristics**	
Age, years (mean ± SD)	27.5 ± 5.59
Urban environment, N (%)	218 (54.3)
Formal education, number of years	
≤ 8 (%)	25.5
9-12 (%)	40.5
> 12 (%)	34
Married, N (%)	323 (80.8)
Number of births, N (%)	
Primiparous	198 (49.5)
Secundiparous	129 (32.3)
Multiparous	73 (18.2)
Wanted pregnancy, N (%)	360 (90)
Gestational age at first prenatal visit, mean ± SD	2.59 ±1.23
First prenatal visit at ≤ 4 months of pregnancy, N (%)	364 (91)
Number of prenatal visits at general practitioner, mean ± SD	4.82 ± 2.54
Number of prenatal visits at obstetrician, mean ± SD	4.76 ± 3.07
Total number of prenatal visits, mean ± SD	9.58 ± 4.88
Nutritional knowledge score, mean ± SD	11 ± 4.8
Advice on diet, YES (N,%)	168 (42)
Advice on breastfeeding, YES (N,%)	334 (83.5)
Use of folic acid (N,%)	192 (48)
Use of iron (N,%)	181 (45.3)
Use of multivitamins (N,%)	272 (68)

### Statistical analysis

Associations between socio-demographic factors, prenatal care, the level of nutritional knowledge and the intake of nutritional supplements were evaluated using the Chi-square test (χ^2^) and binomial logistical regression [24]. Adjusted odd ratios (aORs) and 95% confidence intervals (95% CI) were first determined after adjusting for the number of prenatal visits, then after adjusting for number of prenatal visits and socio-demographic factors (age, area of residence, level of education). The Hosmer-Lemeshow test was used to evaluate the strength of the models created. For all analyses a p value <0.05 was considered significant; all were undertaken using the Statistical Package for Social Science (SPSS) program for Windows Version 13.0 (SPSS 13.0, Chicago, IL, USA).

### Ethical considerations

The study was approved by the ethics committee of the “Grigore T. Popa” University of Medicine and Pharmacy in Iaşi and permission to administer the questionnaires was received from the Cuza-Vodă Obstetrics and Gynaecology Clinical Hospital. Those who participated gave written informed consent. Those younger than 18 years gave their consent in the presence of and with the approval of one of their parents or legal guardians. All data were anonymised to maintain participant confidentiality.

## Results

We found that 48.0% of participants used folic acid, 45.3% used iron supplements and 68.0% used multivitamins during pregnancy (Table 1).

### Socio-demographic characteristics of the study group and description of prenatal care

The mean age of participants was 27.5 years: 63.8% were aged between 19 and 30 years, 5.3% were ≤18 years and 8.8% were aged over 35 years. The majority lived in an urban area (54.3%). Regarding formal education, 25.5% had completed less than 8 years of school, whereas 34.0% had a university degree. The majority of women (80.8%) were married. Primiparous and secundiparous women were predominant (49.5% and 32.3%, respectively); 10.0% of women declared that the pregnancy was unwanted (Table 1).

Most women (96.0%) registered their pregnancy with their general practitioner and had the first prenatal visit in the second (61.0%) or the third (19.0%) month; only 9.0% of women had the first visit in the fifth month. The average number of visits to the general practitioner was 4.8 ± 2.54. The mean number of check-ups with the obstetrician was 4.7 ± 3.07; but 16.5% of women did not see an obstetrician during their pregnancy. The mean total number of combined visits was 9.58 ±4.88: 18% of participants made ≤4 visits, 28.5% made between 5–9 visits and 53.3% had ≥10 visits. Less than half the participants (42.0%) declared they received advice on diet during pregnancy and 83.5% stated they were advised to breastfeed (Table 1).

### Factors associated with vitamin and mineral supplementation during pregnancy

Age, level of education, being married and low gestational age at the first prenatal check-up and total number of prenatal medical visits were positively associated with the use of folic acid, iron and multivitamin supplements. The intake of vitamin and mineral supplements was significantly less common in women who declared the pregnancy was unwanted. Significant differences were noted in the use of folic acid and multivitamin supplements, but not in the use of iron supplements, depending on area of residence (rural versus urban) and parity. Women with a higher level of nutritional knowledge used folic acid, iron and multivitamin supplements more frequently (Table 2).

**Table 2:** Associations between use of supplements and socio-demographic factors, pre-natal care and nutritional knowledge

	**Folic acid**			**Iron**			**Vitamins**		
	**YES**			**YES**			**YES**		
	**N**	**%**	**p**	**n**	**%**	**p**	**n**	**%**	**p**
Urban environment	138	63.6	**<0.001**	171	78.8	0.14	171	78.8	**<0.001**
Rural environment	54	36.4		101	55.2		101	55.2	
Education (number of years)									
≤8	11	5.7	**<0.001**	45	24.9	**<0.001**	43	15.8	**<0.001**
9-12	82	42.7		75	41.4		110	40.4	
> 12	99	51.6		61	33.7		119	43.8	
Age (years old)									
< 18	0	0	**<0.001**	9	42.9	**0.043**	9	42.9	**0.011**
≥ 18	192	50.7		263	69.4		263	69.4	
Number of births									
≤ 2	173	52.6		242	73.6		242	73.6	
> 2	19	26.8		30	42.3		30	42.3	
Wanted pregnancy	179	49.9	**0.017**	254	70.8	**0.009**	254	70.8	<0**.001**
Unwanted pregnancy	12	30.0		17	42.5		17	42.5	
Married	176	54.5	**<0.001**	235	72.8	**0.01**	235	72.8	**<0.001**
Single	16	20.8		37	48.1		37	48.1	
Pregnancy registration									
General practitioner									
Yes	189	49.1	**0.027**	267	69.4	0.**006**	267	69.4	**0.003**
No	3	20		5	33.3		5	33.3	
Obstetrician									
Yes	185	55.4		255	76.3		255	76.3	
No	7	10.6		17	25.8		17	25.8	
Total number or prenatal visits									
≤ 4	2	2.8	**<0.001**	18	25.0	**<0.001**	18	25.0	**<0.001**
> 4	190	57.9		254	77.4		254	77.4	
Gestational age at registration (months)									
<4	180	53.7	**<0.001**	192	57.3	**0.019**	244	72.8	**<0.001**
≥4	12	18.5		27	41.5		28	43.1	
Nutritional questionnaire score									
Low	7	10.1	**<0.001**	23	33.3	**<0.001**	22	31.9	**<0.001**
Medium	137	47.1		147	56.8		192	74.3	
High	48	65.8		49	67.6		58	79.7	

The proportion of less well-educated women (less than 8 years of school completed) using folic acid (p = .007), iron (p < .001) and multivitamins (p < .001) increased with the number of prenatal visits. The same relationship was observed for women with 9–12 years of formal education regarding folic acid use (p < .001), iron (p = .001) and multivitamins (p < .001). There was no significant difference of using folic acid, iron and multivitamin supplements in relation to the number of prenatal visits, for women with higher education level (Table 3). When analysing separately the interaction effect between education and prenatal care, the probability of using folic acid, iron and vitamins supplements was increasing with the level of formal education and number of prenatal visits (Table 4).

**Table 3:** Associations between use of folic acid, iron and multivitamin supplements and number of prenatal visits, among categories of formal education

**Formal education (years)**	**Number of prenatal visits**			**p**
	**<4**	**4-10**	**>10**	
**Folic acid use (%)**				
**≤8**	2.1	14.6	30.8	.007
**9-12**	4.3	44.3	72.5	<.001
**>12**	0	69.4	74.7	.21
**Iron use (%)**				
**≤8**	16.7	56.1	76.9	<.001
**9-12**	21.7	51.4	66.7	<.001
**>12**	100	77.8	62.6	.19
**Multivitamins use (%)**				
**≤8**	22.9	53.7	76.9	<.001
**9-12**	26.1	70	79.7	<.001
**>12**	100	88.9	87.5	.88

**Table 4:** Associations between use of folic acid, iron and multivitamin supplements and the interaction between the number of prenatal visits and formal education level

		**Folic acid**		**Iron**		**Vitamins**	
		**OR (95% CI)**	**OR**^**# **^**(95% CI)**	**OR (95% CI)**	**OR**^**# **^**(95% CI)**	**OR (95% CI)**	**OR**^**# **^**(95% CI)**
Education*Prenatal care interaction	PC1*E1	110.63 (14.94-818.97)	63.86 (8.47-481.41)	10.35 (4.60-23.27)	7.95 (3.36-18.83)	18.20 (8.46-39.16)	11.25 (4.96-25.49)
	PC1*E2	51.78 (6.83-392.29)	32.281 (4.97-294.63)	7.45 (2.66-20.88)	6.20 (2.17-17.73)	15.33 (5.64-41.68)	11.71 (4.17-32.87)
	PC2*E1	13.73 (5.50-34.22)	10.941 (4.30-27.78)	1.62 (0.82-3.19)	1.38 (0.68-2.79)	4.67 (2.28-9.55)	3.65 (1.72-7.71)
	PC2*E2	2.96 (1.70-5.15)	2.51 (1.41-4.49)	1.95 (1.13-3.38)	1.73 (1.10-3.03)	2.31 (1.23-4.34)	2.03 (1.05-3.91)
**Nutritional knowledge score**	Low	1	1	1	1	1	1
	Medium	6.67 (2.62-17.01)	1.47 (0.74-2.89)	1.80 (0.99-3.27)	1.65 (0.86-3.15)	3.58 (1.88-6.80)	1.14 (0.52-2.50)
	High	8.30 (2.99-23.4)	7.13 (2.33-21.80)	2.41 (1.14-5.14)	2.37 (1.50-5.65)	3.48 (1.50-8.10)	3.62 (1.38-9.51)

PC1 – prenatal care:≥ 10 visits;PC2 - prenatal care: 4–9 visits; reference category - < 4 visits.

E1 – education: >12 years; E2 – 9–12 years; reference category - ≤ 8 years.

OR^#^-adjusted for the interaction effect (PC*E).

After adjusting for the total number of prenatal visits, we noted independent effects of area of residence and level of education on the use of supplements: women from urban areas and those with more than 9 years of formal education were more likely to have used folic acid (urban area: aOR, 1.5; 95% CI, 1.2-1.5; 9–12 years of education: aOR, 4.8; 95% CI, 2.3-10.0; >12 years of school: aOR, 8.1; 95% CI, 3.7-18.0) and multivitamin supplements (urban area: aOR, 1.7; 95% CI, 1.0-2.7; >12 years of school: aOR, 3.5; 95% CI, 1.7-7.4) (Table 5). However these socio-demographic factors were not independent determinants of the use of iron supplements.

**Table 5:** Determinants of the use of folic acid, iron and vitamins

	**Folic acid**		**Iron**		**Vitamins**	
	**OR (95% CI)**^**#**^	**OR (95% CI)**^**##**^	**OR (95% CI)**^**#**^	**OR (95% CI)**^**##**^	**OR (95% CI)**^**#**^	**OR (95% CI)**^**##**^
Urban environment	**1.47 (1.18;1.48)**	1.46 (0.83; 2.54)	0.86 (0.55; 1.34)	0.72 (0.44; 1.19)	**1.66 (1.01; 2.73)**	1.58 (0.90; 2.78)
Age (yrs)						
≤19	1	1	1	1	1	1
19-34	2.06	1.27	1.05	0.93	1.55	1.18
	(0.66;6.47)	(0.39;4.19)	(0.47;2.37)	(0.41;2.14)	0.67;3.58	0.50;2.78
≥35	3.11	1.59	0.76	0.64	0.79	0.50
	(0.84;11.46)	(0.41;6.17)	(0.28;2.08)	(0.23;1.79)	0.28;2.25	0.17;1.47
Schooling (grades)						
≤ 8						
9-12	**1**	**1**	1	1	1	1
	**4.76**	**4.01**	1.14	1.34	1.63	1.640
>12	**(2.28;9.97)**	**(1.88;8.56)**	(0.66;1.98)	(0.75;2.37)	(0.93;2.88)	(0.908;2.964)
	**8.12**	**5.21**	1.54	**2.30**	**3.54**	**3.372**
	**(3.67;17.96)**	**(2.12;12.82)**	(0.82;2.90)	**(1.08;4.86)**	**(1.68;7.43)**	**(1.432;7.940)**
Married	**2.68**	1.93	1.30	0.74	1.49	1.330
	**(1.34;5.36)**	(0.93; 4.01)	(0.75;2.25)	(0.45;1.22)	(0.83;2.68)	(0.712;2.487)
Wanted pregnancy	0.96	0.82	1.61	1.48	0.96	1.49
	(0.40;2.30)	(0.32; 2.08)	(0.78;3.33)	(0.71;3.09)	(0.40;2.30)	(0.68;3.26
First birth	1.354	0.947	0.913	0.898	2.036	1.79
	(0.706;2.599)	(0.474;1.891)	(0.519;1.604)	(0.506;1.593)	(1.128;3.673)	(0.98;3.26)
≤4 pregnancy registration	**3.76**	**3.41**	1.07	0.92	0.48	2.02
	**(1.19;11.89)**	**(1.05;11.14)**	(0.52;2.24)	(0.48;1.93)	(0.22;1.05)	(0.91;4.46)
No. check-ups						
<4						
4-9	**1**	**1**	**1**	**1**	**1**	**1**
	**18.92**	**12.06**	**5.70**	**5.55**	**5.34**	**4.12**
≥10	(4.41;81.24)*	(2.75;52.84)**	(2.85;11.38)*	(2.70;11.39)**	(2.77;10.29)*	(2.08;8.15)**
	**83.03**	**35.92**	**7.77**	**7.16**	**16.44**	**9.38**
	(19.5;344.7)*	(8.24;156.5)**	(4.02;14.67)*	(3.42;15.00)**	(8.59;31.51)*	(4.50;19.48)**
Low score	**1**	**1**	**1**	**1**	**1**	**1**
Medium score	**6.67**	**4.43**	**1.80**	**1.79**	**3.58**	**3.08**
High score	**(2.62;17.01)**	**(1.70;11.55)**	**(0.99;3.27)**	**(0.95;3.35)**	**(1.88;6.80)**	**(1.58;6.01)**
	**8.30**	**4.72**	**2.41**	**2.58**	**3.48**	**2.81**
	**(2.99;23.40)**	**(1.61;13.84)**	**(1.14;5.14)**	**(1.16;5.74)**	**(1.50;8.10)**	**(1.16;6.84)**
Breastfeeding advice	1.85	1.50	1.96	1.97	1.26	1.10
	(0.96;3.55)	(0.76;2.95)	(1.12;3.46)	(1.11;3.50)	(0.68;2.32)	(0.59;2.06)
Diet advice	1.53	1.39	**1.56**	1.53	**1.71**	1.59
	(0.96;2.44)	(0.85;2.27)	**(1.01;2.40)**	(0.98;2.37)	**(1.03;2.84)**	(0.95;2.68)

*: crude OR; **: OR adjusted for age, schooling, residence area.

#: adjusted OR for total number of prenatal medical visits.

##: adjusted OR for total number of prenatal checks, age, schooling, residence area. With bold significant OR for p<0.005.

Women who stated that they had received dietary or breastfeeding advice had a higher probability of using folic acid or multivitamins regardless of the number of prenatal visits. Early pregnancy registration was significantly associated with the intake of folic acid (aOR, 3.8; 95% CI, 1.2-11.9). Primiparous women were more likely to use multivitamins regardless of the number of prenatal visits (aOR, 2.0; 95% CI, 1.1-3.7). Making more than four prenatal visits was also associated with folic acid, iron and multivitamin supplements intake, a positive association that persisted after adjusting for socio-demographic factors (Table 5).

An above average level of nutritional knowledge was significantly associated with the use of supplements, regardless of the number of prenatal consultations, age, area of residence or formal education (Table 5), but also when the interaction effect of prenatal care and formal education together was taken into consideration (Table 4).

## Discussion

In our study, less than half the participants used folic acid or iron during their pregnancy, while approximately two thirds took multivitamin supplements. Nutritional knowledge was significantly associated with the intake of these supplements, regardless of the frequency of prenatal visits or socio-demographic characteristics. To the best of our knowledge, this is the first study to have examined the factors that influence use of vitamin and mineral supplements during pregnancy in Eastern Europe.

The use of supplements during pregnancy varies widely between countries. These differences cannot be compared directly due to discrepancies in national guidelines and the financial barriers in some regions [27]. The most popular vitamin and mineral supplements contain iron, folic acid and vitamin D [12,28].

Routine use of iron supplements is common, as it is difficult to ensure adequate intake from food sources, even from those with relatively high iron bioavailability [29,30]. The Institute of Medicine recommends a daily intake of 400 μg of folic acid and 30 mg of iron during the second and third trimesters [31]. Vitamin and mineral supplements are recommended for women with multiple pregnancy, those who misuse alcohol or drugs, or have an unbalanced diet [32]. The World Health Organization recommends a daily intake of 60 mg iron and 400 μg folic acid for six months to prevent anaemia [33].

Romanian recommendations stipulate that women with a haemoglobin higher than 11 g/dl should take 60 mg of iron and 350 μg of folic acid from the 16^th^ week of pregnancy to prevent anaemia, but only iron products are reimbursed by the health insurance system. To prevent neural tube defects, 400 μg of folic acid should be taken daily, starting one month before conception and throughout the first trimester. The routine use of multivitamins is not recommended [34]. We evaluated the determinants for the intake of multivitamins as previous studies, as well as the common practice in Romania suggested a high incidence of use [14,35], despite the lack of recommendation.

We found that 48.0% of women took folic acid at some stage during pregnancy, compared with 27.0% in the IOMC study. This may have arisen as a consequence of the IOMC study only examining folic acid use during the last trimester of pregnancy [14]. The proportion of women in our study group taking folic acid is still low, despite its widely recognised benefits, but could be explained by the lack of reimbursement for the cost of folic acid, which must be bought over the counter at any time it is prescribed, during pregnancy or before conception.

The reported adherence rates to folic acid intake in other countries vary depending on concomitant recommendations for cereal fortification and on the period analysed – pre- or peri-conception. In a meta-analysis undertaken by Pena-Rosas et al. [32], the proportion of women taking folic acid varies between 0.9% and 49% before conception and between 0.5% and 52% in the peri-conception period. Our results lie within these ranges, but it is difficult to make a comparison, as the variations are very wide and we recorded the use of folic acid throughout pregnancy without focusing on the first trimester. It could be argued that as routine prenatal care in Romania does not usually begin until after the first trimester is almost ended, including advice about folic acid as part of prenatal care is redundant as the neural tube has fully developed within 28 days of conception [9]. Nonetheless, folic acid supplementation might address other forms of anaemia, and the advice might also increase the likelihood of folic acid use before and during future pregnancies.

Among the factors evaluated in our study, area of residence, level of education and marital status were associated with the intake of folic acid during pregnancy, independently of the number of prenatal visits. This observation could be a consequence of social inequity or particular health beliefs. The number of prenatal visits, registration with a physician early in pregnancy and the level of nutritional knowledge were independently associated with the use of folic acid, after adjusting for socio-demographic factors, suggesting that educational interventions could play an important role in promoting the use of folic acid supplements. The observation that early registration with a physician in pregnancy was associated with folic acid intake could indicate the recognised importance of early prenatal care. Furthermore, it is perhaps unsurprising that women with higher educational achievement and also those with greater engagement with prenatal care had better nutritional knowledge and used supplements more. Importantly, we also found that less well-educated women who engaged with prenatal care were more likely to take folic acid. This suggests that the interventions (prenatal visits) are productive, and that the Romanian government should seek ways of encouraging less well-educated women to participate.

Some of our findings are comparable with those of studies conducted in other countries. Low adherence with advice about supplements was associated with unwanted pregnancy, low maternal age and low economic status in the United Kingdom [36], unplanned pregnancy and lack of education regarding the role of folic acid in South Korea [37]. The impact of nutritional knowledge on the use of folic acid was emphasised in a study conducted in Turkey, where factors negatively associated with pre-conception supplementation were low socio-economic status, age below 30 years, low levels of education and unplanned pregnancy. The same study stressed that the level of knowledge about food sources and the role of folic acid during pregnancy was influenced by socio-demographic factors, especially the level of education [38]. In our study, the level of nutritional knowledge was an independent determinant for the use of folic acid during pregnancy.

A relatively low proportion of women in our study used iron supplements (45.3%), although these are provided free of charge to women after the 16^th^ week of pregnancy. A previous study in Romania reported that supplemental iron was taken by 53.3% of women during pregancy [14]. A Danish study reported that adherence with an iron supplementation regime was associated with maternal age (over 20 years), primiparity and educational level [18]. These results are similar to ours: we found that women aged over 18 years, with parity less than two, with more than 9 years of education, from urban areas with a wanted pregnancy or who were married were significantly more likely to use iron supplements. After adjusting for socio-demographic factors, more than four prenatal visits and level of nutritional knowledge remained associated with iron supplementation. This highlights the importance of prenatal care, both in terms of frequency and content, in improving adherence to programs to prevent anaemia.

Multivitamins were used by 68% of the women in our study, comparable with the 60% reported by the IOMC study [14]. This is a surprisingly high proportion, especially when there is no strong recommendation for the use of multivitamins in Romania or internationally. Moreover, reimbursement for multivitamin supplements is not covered by insurance in Romania, suggesting that there are other factors contributing to their use. Some studies describe a positive association between the use of multivitamins during pregnancy and certain socio-demographic characteristics, such as age [15,16,18,20], education [15,17,18,36,39] and marital status [17]; but others have reported an inverse relation between education and the use of multivitamins [27]. There is also evidence of a relationship between engagement with prenatal care and use of multivitamin supplements, the adequacy of prenatal care being assessed according to Adequacy of Prenatal Care Utilisation Index, which takes into account the month of initiation of care and the proportion of recommended visits adjusted for the gestational age at initiation of care and gestational age at delivery [27]. This index is not recorded in Romania; instead, we evaluated engagement in prenatal care according to Ministry of Health recommendations, which refer to the total number of prenatal consultations and time of initiation of care. The content of prenatal care, including nutritional education, is an important factor that could determine the use of supplements during pregnancy, but women may also receive advice from friends, family, the media and other sources.

In our study, women who scored at least “medium” in the nutritional knowledge questionnaire were more likely to use multivitamins. Thus, even though there is no consensus on recommending the use of multivitamins during pregnancy, they are used more often by those women with a better socio-economic status, with a higher level of education and who attended more prenatal consultations. Other studies show that factors associated with multivitamin supplementation during pregnancy are schooling level [15,40] and age [28]. The results of the Behavioural Risk Factor Surveillance System (BRFSS) also emphasize the role of income and marital status in the use of vitamin and mineral supplements [40].

In our study, after adjusting for the number of prenatal consultations, the only socio-demographic factors that persisted on being associated with the use of multivitamins during pregnancy were: area of residence; level of education; parity and receipt of advice about diet and breastfeeding. This might suggest the existence of social inequities and the influence of economic factors in making health-related decisions, as in our country, women in the urban area, more educated and with less children are usually having a better financial status. After adjusting for socio-demographic factors, making more than four prenatal consultations remained associated with the use of multivitamin supplements. These supplements are not necessary in most pregnancies, which raises questions about women’s motivation to use them in the absence of formal recommendations. Level of nutritional knowledge was associated with the use of multivitamins regardless of the number of prenatal visits, age, area of residence and level of education, which could suggest that the decision to use them depends on other factors, such as attitudes towards pregnancy or beliefs about the importance of vitamins during pregnancy, areas that warrant further study.

### Limitations

This study is based on retrospectively self-reported data, which could have led to an underestimation of the use of supplements, especially folic acid [27]. We mitigated against this possible source of error by asking additional questions about the type of supplement and its commercial name. This was the first use of a newly developed questionnaire, which we administered 2–3 days after delivery, to include women who may not have engaged with prenatal care. Having done so, however, some of our participants may not have accurately remembered their use of supplements early in pregnancy or before conception.

Even though our results highlight the importance of nutritional knowledge in the decision to use iron or folic acid, we cannot draw conclusions about overall compliance with supplement regimes, because we did not record the dose or duration of iron, folic acid or multivitamin use. Furthermore, we did not record when during the pregnancy the supplements were taken, which is of particular importance for folic acid. The sample size of our study was determined based on an unknown prevalence of supplements intake (which was set by default to be 50%), but may have been too small to accommodate some subgroup analyses; we must therefore be cautious about the conclusions that can be drawn from subgroup analysis and test the hypotheses that arise from these findings in future studies.

As this is an observational study, we cannot conclude that the information about the roles or sources of nutrients or general recommendations about diet during pregnancy directly influence the frequency with which supplements are used, despite the fact that nutritional knowledge appears to be an important determinant. Additional studies are required better to understand women’s attitudes towards using supplements and the way nutritional knowledge is delivered and used.

## Conclusions

The level of nutritional knowledge was significantly and independently associated with iron, folic acid and multivitamin supplements use during pregnancy. Engagement with prenatal care appeared to increase the likelihood that less well-educated women would take supplements during pregnancy, suggesting that the prenatal nutritional advice may be a useful intervention. From a public health perspective, this is crucially important, and should motivate more effective implementation of nutritional educational programmes for women of childbearing age.

## Competing interests

The authors declare that they have no competing interests.

## Authors’ contributions

All authors listed have contributed sufficiently to the project to be included as authors, and all those who are qualified to be authors are listed in the author byline. ADP, ON, LM, and MG contributed to the study conceptualization and design, acquisition of data, analysis and interpretation of data and writing of article. ADP, GEB and LIG contributed to study conceptualization and design, analysis and interpretation of data and writing the article. RMP and MG commented on the manuscript. All authors have read and approved the submitted manuscript.

## Pre-publication history

The pre-publication history for this paper can be accessed here:

http://www.biomedcentral.com/1471-2458/13/1105/prepub

## Supplementary Material

Additional file 1 Nutritional knowledge questionnaire. Click here for file
