# Supplementary material for: Nutritional knowledge as a determinant of vitamin and mineral supplementation during pregnancy
Source: BMC Public Health. 2013 Dec 1;13:1105. doi: 10.1186/1471-2458-13-1105 (PMC3933411; doi:10.1186/1471-2458-13-1105)
Supplement: Additional file 1 — Nutritional knowledge questionnaire. [file 1471-2458-13-1105-S1.pdf]

Additional file 1. Nutritional knowledge questionnaire

	Question	Answer		
		yes	no	Don't know
1	During pregnancy a varied diet is recommended			
2	A pregnant woman should not eat for two			
3	During pregnancy it is recommended to eat bread, cereal, vegetables and fresh fruit			
4	The recommended dairy quantity during pregnancy is 600 grams.			
5	During pregnancy it is recommended to increase the consumption of proteins			
6	The recommended weight gain during pregnancy is 9-12,5 kg.			
7	Pregnant women should eat 4,5 times a day			
8	Food with quality proteins are: meat, eggs, milk			
9	Healthy fats are from : sunflower oil, soya, olives			
10	Food rich in iron are: eggs, meat, spinach, liver, beans			
11	Food rich in folic acid are green vegetable			
12	Food rich in calcium are: milk, fish, green vegetables			
13	Folic acid is important in the development of the nervous system.			
14	A diet lacking iron leads to anaemia			
15	Calcium is important in the growth of teeth and bones.			
16	Iodine is important for the intellectual development of the child			
17	Alcohol is damaging during pregnancy			
18	Breast milk is the best nourishment for the child.			
19	Lack of iron in the baby's diet leads to anaemia.			
20	Anaemia appears in babies fed pasta and the like.			
21	Lack of vitamin D leads to rickets.			
22	A diet lacking proteins slows down growth.			
23	In the first 6 months a diet of breast milk only is recommended.			
24	Breast milk provides an adequate quantity of energy and nutrients.			
25	Breast milk protects the baby from infections.			
26	Decreasing the calorie input leads to decreased lactation.			
27	Protein consumption should be increased during lactation.			
28	While breastfeeding the consumption of 2-3 litres of liquids a day is recommended.			
29	Beer consumption increases the quantity of milk.			
